# A Cognitive-Inspired Event-Based Control for Power-Aware Human Mobility Analysis in IoT Devices

**DOI:** 10.3390/s19040832

**Published:** 2019-02-18

**Authors:** Rafael Pérez-Torres, César Torres-Huitzil, Hiram Galeana-Zapién

**Affiliations:** 1 CINVESTAV-Tamaulipas, Ciudad Victoria C.P. 87130 Tamaulipas, Mexico; hgaleana@tamps.cinvestav.mx; 2 Tecnologico de Monterrey, School of Engineering and Sciences, Campus Puebla, Av. Atlixcayotl 5718, Puebla C.P. 72453 Puebla, Mexico; torresc@tec.mx

**Keywords:** trajectory, stay point, cognitive control, smartphone, location, power-aware

## Abstract

Mobile Edge Computing (MEC) relates to the deployment of decision-making processes at the network edge or mobile devices rather than in a centralized network entity like the cloud. This paradigm shift is acknowledged as one key pillar to enable autonomous operation and self-awareness in mobile devices in IoT. Under this paradigm, we focus on mobility-based services (MBSs), where mobile devices are expected to perform energy-efficient GPS data acquisition while also providing location accuracy. We rely on a fully on-device Cognitive Dynamic Systems (CDS) platform to propose and evaluate a cognitive controller aimed at both tackling the presence of uncertainties and exploiting the mobility information learned by such CDS toward energy-efficient and accurate location tracking via mobility-aware sampling policies. We performed a set of experiments and validated that the proposed control strategy outperformed similar approaches in terms of energy savings and spatio-temporal accuracy in LBS and MBS for smartphone devices.

## 1. Introduction

Smart devices in IoT systems incorporate multiple sensors that potentially enable them with context-awareness. Among these sensors, location sensors play a critical role in IoT, specifically in use cases in which nodes, such as smartphones, are dispersed on large areas and might have different mobility patterns. For these cases, continuous fine-grained location readings are fundamental for further individual mobility pattern mining. However, energy consumption deters continuous location sensing as embedded GPSs are power-hungry modules with a high cost on battery-powered devices. This calls for novel solutions to reduce energy consumption without compromising the quality of location data in terms of accuracy and granularity. In particular, we have recently witnessed a paradigm shift from Mobile Cloud Computing (MCC) toward Mobile Edge Computing (MEC) fog computing solutions, which also contribute to reducing the latency and enhance the security of collected information: mobile devices are expected to play a more active processing role rather than simply sensing and transmitting tasks [1].

An individual’s mobility can be modeled as a sequence of events raised from the interaction with frequent places [2]. A mobility sensing application might take advantage of these events and their patterns to adjust the GPS sampling accordingly and to avoid redundant location fixes to save energy (power-aware event-based sensing). Instead, periodic sampling does not take into account the diversity in actual human mobility. Thus, for a better energy control in always-on and power-aware on-device location and mobility sensing, it is desirable to [3]: (i) take into account adaptive sampling rate of the employed sensors and (ii) implement optimized local analytic and control mechanisms to assist this purpose.

In this regard, advances in neuroscience offer control engineering a source of inspiration to recast classic controllers into more optimized and adaptive ones, based on the claim that machines and living organisms might exhibit similar behavioral mechanisms, with parallels between computation and control mechanisms [4,5]. Cognitive control relies on the concept of the *information gap* between what a Cognitive Dynamic System (CDS) knows about the environment and what it must know to achieve a goal. It is inspired by the *action-oriented theory of controlled sensing*: what we perceive is determined by what we do [5], so that the relevance of actions is only determined within the context of perception in an environment prone to unexpected and unpredictable events [6]. However, the successful application of CDSs to real-world problems in mobile devices has still been rather limited [7].

Under event-based sensing and CDS paradigms, in a previous work [7], we proposed an on-device sensing architecture, without cloud-dependency, to characterize an individual’s mobility based on mobility events. In this paper, we extend our previous work and propose a cognitive controller that exploits mobility information learned by a CDS for accurate and energy-efficient on-device location tracking via mobility-aware sampling policies.

We argue that CDS-inspired solutions are fundamental to enable a truly distributed intelligence of mobile devices, a highly desirable feature in IoT systems. Similarly, we stress that event-based control, where GPS sampling is event-triggered, is closer in nature to how humans interact with the environment, and it suits a clear purpose for mobile devices with proven control performance and low resource utilization [8]. Our main contribution is summarized as follows: an innovative power-aware control approach for IoT devices to enable on-device location data processing and adaptive GPS sampling rates, based on cognitive control and event-based processing. We experimentally demonstrate that a good accuracy-energy tradeoff for continuous location tracking is achieved by the proposed cognitive controller, where associations between perception and action are formed via the individual’s interaction with the environment.

The rest of this article is structured as follows. Section 2 presents the related work for location data processing toward energy efficiency. Section 3 describes the proposed event-based representation of an individual’s mobility. Section 4 details our cognitive controller strategy for accurate power-aware location and mobility tracking. The implementation of our solution and its experimental evaluation are discussed in Section 5, followed by conclusions in Section 6.

## 2. Related Work

Bio-inspired cognitive control encompasses the collection of processes that are involved in generating and maintaining appropriate task goals (suppressing those no longer relevant), as well as the way in which the current goal is used to modify attentional biases to improve task performance [9]. As proposed by Haykin [4,5], cognitive control implies a perception-action cycle, on which the recently-perceived stimuli (in conjunction with learned information across time) have an impact on the decisions made by the controller. Cognitive control is present in the decision making of multiple human daily tasks, for instance in word prediction while reading [10], and in general, it allows the individual to adapt when conflicts or contradictions with previous experience and expectations emerge.

Given its importance toward behavior understanding, a rich body of research has been carried out toward human activity recognition. Sources such as raw inertial sensor data [11] and video analysis have been employed for identifying and even modeling the transitions between the elemental steps of complex activities like cooking or playing basketball [12].

Correctly modeling and understanding user behavior helps the better employment of computing, energy, and sensing resources of a mobile device [2]. Regarding user’s mobility, prior works have investigated energy efficiency in smartphone-based sensing platforms from different perspectives, as it is essential for application and system developers to maximize the smartphone’s battery lifetime. As surveyed in [13], there are multiple strategies for making the most of the mobile battery when employing different smartphone components. For mobility sensing, in [14], energy-efficient strategies for continuous sampling in Location-Based Services (LBSs) were analyzed: approaches such as duty cycling, recycling of existing data, and next data prediction have been documented.

Rather than describing the particularities of the proposed solutions (see existing surveys [13,14,15]), herein, we focus our discussion on their high level characteristics. Most of the solutions sort the *energy consumption vs. accuracy* tradeoff by adjusting the duty cycle of the GPS [16,17] or by replacing it with another less expensive energy location provider (cellular ID, Wi-Fi Positioning System (WPS)) [18,19,20]. Similarly, some solutions replace the GPS with sensors that indirectly provide information of the user’s displacements [21,22,23], aiming to account for the traveled distance in sampling rate adjustments. Nevertheless, the data analysis in such solutions usually refers to short-term or instantaneous mobility; adaptations are thus not tied to representative spatio-temporal characteristics of human mobility, but only to recent speed changes. In other words, the understanding of an individual’s mobility has received little attention for on-device exploitation toward energy efficiency.

On the other hand, the availability of large amounts of location data calls for techniques for individual and collective human mobility mining [24], which gives a high-level meaning to raw location data, making possible Mobility-Based Services (MBSs) such as place recommendation, route suggestions, and mobility prediction. MBSs, unlike LBSs, which react to changes in raw location data, self-adapt to people mobility patterns.

The extraction of mobility information in MBSs has been typically performed using an MCC approach [25], where the smartphone only collects and transmits location data to be processed in external computing entities (the cloud) to achieve a degree of mobility understanding. An MCC approach is suitable for community sensing [15] to concentrate mobility information of groups of people, but it faces important issues like latency, data fees, and energy consumption derived from raw data transmission [1,26]. In any case, the analysis of an individual’s mobility does not require such a concentration of location data, so that it can be performed directly on-device, the paradigm followed in this work.

Nonetheless, a limited number of works have been proposed for on-device mobility mining. An example of such on-device solutions is the SmartDC framework [27], which collects cellular ID and WPS data to learn user’s frequent places (semantically, rather than raw coordinates) and to predict stay times to reduce the sensors’ sampling rate following an optimization approach. However, a drawback of optimization-based solutions is that the underlying modeling of sensors’ accuracy, energy consumption, energy budget, and battery characteristics might not be held in practice. This is because the operation of smartphone components can differ from a given modeling (accuracy changes, lack of sensors data, non-linear battery discharge, etc.), which in the best scenario requires the sampling rate to be constantly updated to become feasible. Additionally, optimization parameters (expected running time, energy budget, accuracy level, etc.) might be hard to specify and tune for the diversity of mobility conditions and scenarios that could be found in LBSs [14].

We argue that optimization approaches can be enriched with spatio-temporal attributes of the user’s mobility. Thus, we propose a cognitive controller that reduces the uncertainty about user’s mobility using adaptive sampling policies tailored to the current mobility state. Such policies are implemented only when major changing events are detected.

## 3. Individual Mobility Modeling

**Figure 1:**
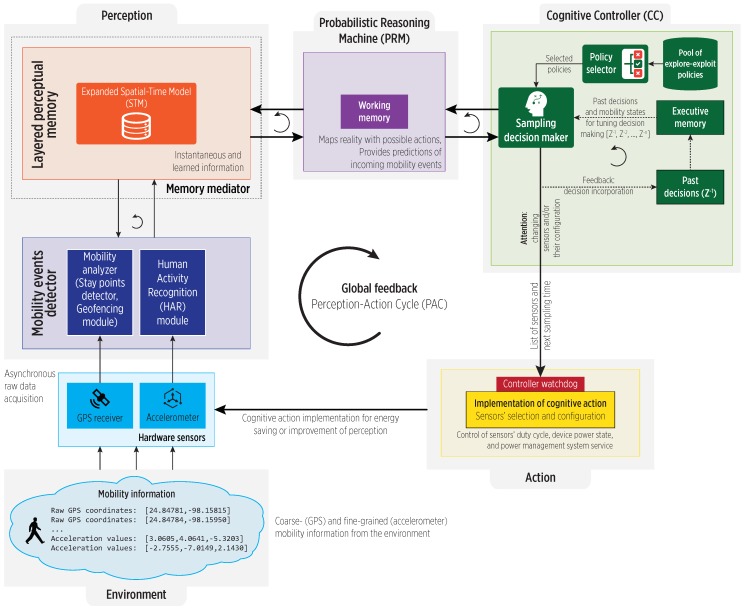
Architecture of our Cognitive Dynamic Systems (CDS) [2] for mobility characterization and events’ prediction.

In our work, stay points are the cornerstone for characterizing an individual’s mobility in a spatio-temporal model (cognitive map) [2], under the simplified CDS architecture shown in Figure 1. The architecture is structured into three main components: the perceptor that perceives the surrounding environment and builds an internal representation of it, the actuator (controller) that generates actions on the environment, and the working memory (in a Probabilistic Reasoning Machine (PRM)) that provides a dynamic coupling between perception and action. These components form a closed-loop feedback system, i.e., the perception-action cycle. The structure of the cognitive map is a multigraph, on which nodes refer to stay points (spatial information) and edges represent the user’s visit to them (temporal attributes).

**Figure 2:**
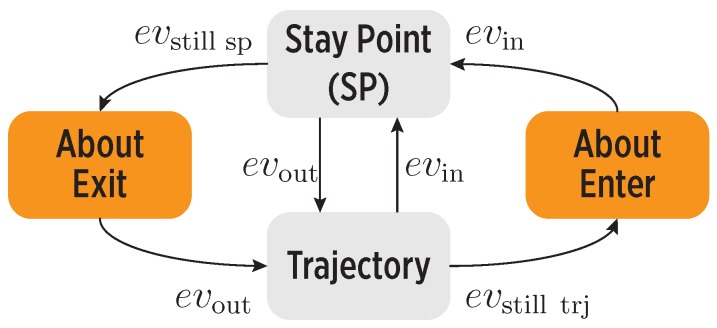
The mobility states in the cognitive controller and the transitions between them caused by mobility events. An enter event ($ev_{\mathrm{in}}$) causes the system to reach the $S_{\mathrm{SP}}$ state, indicating that the user arrived to a previously-known place. An exit event ($ev_{\mathrm{out}}$) leads to the $S_{\mathrm{trajectory}}$ state, denoting that the user left a place and headed to the next one. The user might not follow the learned mobility patterns: when the user does not leave a place at the expected time, an $ev_{\mathrm{still}\;\mathrm{sp}}$ event moves the system to the $S_{\mathrm{about}\;\mathrm{exit}}$ state; when the user keeps on a trajectory and does not arrive at a place as expected, an $ev_{\mathrm{still}\;\mathrm{trj}}$ event makes the system reach the $S_{\mathrm{about}\;\mathrm{enter}}$ state.

We model an individual’s mobility as a sequence of states, as shown in Figure 2. The transitions between states are handled by the working memory of our system, based on the input stimuli and the learned mobility in the map, e.g., the regularity of individual’s routines. Besides enter and exit events, there are also events linked to no changes in mobility, i.e., the user is still inside a stay point ($ev_{\mathrm{still}\;\mathrm{sp}}$) or in a trajectory ($ev_{\mathrm{still}\;\mathrm{trj}}$). Such events are relevant to detect mobility inconsistencies, as described later on.

### 3.1. Mobility States

The working memory derives meaningful information from the current mobility state and transitions (see Figure 2), for decision making in the cognitive controller. Table 1 provides a brief description of these states; notice that we define two types of mobility states:

- **Observed mobility states**: They are detected by system perceptors without involving further processing, i.e., the $S_{\mathrm{SP}}$ and $S_{\mathrm{trajectory}}$ states.
- **Derived mobility states**: They are detected by the working memory when inconsistencies (mismatches) are found between the observed mobility and the cognitive map information, i.e., the $S_{\mathrm{about}\;\mathrm{enter}}$ and $S_{\mathrm{about}\;\mathrm{exit}}$ states.

### 3.2. Motion States Transition

Human mobility might be expressed in terms of pauses when visiting stay points and the speed during trajectory. On the one hand, a visit to a stay point yields a *mobility state transition* from high to low speed (idleness) during enters and low to high speed during exits. Considering that a high speed requires a high sampling rate to ensure spatial accuracy, then a high rate would be appropriate at the enter and exit stay points. On the other hand, a low sampling rate at the middle of a visit might suffice as motion is not expected to occur. A sampling rate that accounts for such a motion state transition could produce a spatio-temporal accurate tracking while also achieving energy efficiency.

Similarly, when moving between stay points, the transportation mode, detected by a Human Activity Recognition (HAR) module, is used to infer the user’s speed to adjust the GPS sampling rate, again assuming a fast rate for high speeds. We focused on transportation modes in the set {static (**0–1 m/s**), walking (**1–2 m/s**), cycling (**2–5 m/s**), vehicle (**5–20 m/s**, or more)}. The values shown in parenthesis, which are not definitive and can be configured, aid in obtaining such speed insight, as they are typical in people’s mobility [28]. We follow this rationale aiming to cover the different stages of a user’s mobility: the varying motion between stay point displacements and the transition to idleness during stay point visits.

### 3.3. Predictions of Mobility States

The CDS working memory generates spatio-temporal predictions from enter/exit events using the cognitive map information. The timestamp *t* of an event aids the traversal of the cognitive map nodes, filtering out only those transitions meaningful to a prediction, expressed as ρ=(sp,t), where sp is the stay point involved in the predicted event. For instance, whenever the user enters a place (i.e., a $ev_{\mathrm{in}}$ event is detected), the working memory predicts an exit $\rho_{\mathrm{out}}$; similarly, once the user leaves a place ($ev_{\mathrm{out}}$), an enter prediction $\rho_{\mathrm{in}}$ is produced. Predictions aid the cognitive controller in producing actions (i.e., GPS sampling rate adjustments) and detecting irregularity in the individual’s mobility.

### 3.4. Mismatches

A prediction not held indicates that the individual is not moving as expected, so that the system detects a mismatch (inconsistency) and transitions to a derived mobility state. For a prediction ρ and a no-change event ev ($ev_{\mathrm{still}\;\mathrm{sp}}$, $ev_{\mathrm{still}\;\mathrm{trj}}$), a mismatch is detected if:

(1) $$\left|ev.t-\rho .t\right|>\rho_{\mathrm{time}}$$

where $\rho_{\mathrm{time}}$ is a time threshold that adjusts the sensitivity to temporal inconsistencies.

Thus, if an $ev_{\mathrm{still}\;\mathrm{sp}}$ or an $ev_{\mathrm{still}\;\mathrm{trj}}$ event represents a mismatch, the CDS moves to the $S_{\mathrm{about}\;\mathrm{exit}}$ and $S_{\mathrm{about}\;\mathrm{enter}}$ states, respectively. Mismatches are notified to the controller for implementing a sampling policy aimed at improving the CDS perception.

## 4. Cognitive Control for Power Savings

**Figure 3:**
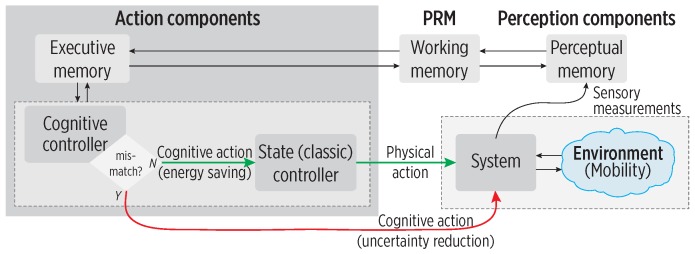
A Cognitive Controller (CC) within a CDS architecture, adapted from [5]. A classic controller produces a physical action if observed mobility is consistent with learned mobility (green arrow). Uncertainty is reduced by increasing the observations in perception (red arrow).

In engineering terms, a Cognitive Controller (CC), within a CDS architecture as shown in Figure 3, holds two main objectives: one is a system-specific objective, while the second one is to reduce the information gap (uncertainty) between the observed information and the sufficient information to achieve the system-specific goal [5]. In our CDS, the CC first detects whether a mobility mismatch exists to implement one of such objectives:

- **To reduce energy consumption**: If consistent mobility is found (low uncertainty), then it is possible to rely on the predictions for adjusting the sampling, i.e., the controller reduces the sensors’ sampling rate to save energy (green arrow).
- **To reduce mobility uncertainty**: If inconsistent mobility is found (high uncertainty due to mobility mismatch), the controller must reduce the information gap by using a sampling focused on improving the mobility perception (red arrow).

**Figure 4:**
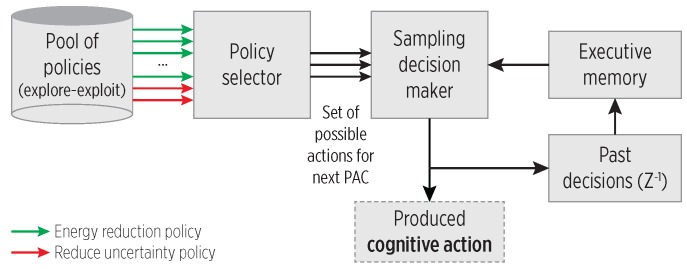
The functional blocks of the proposed CC, adapted from [5]. A pool of explore-exploit policies provides the sampling decision maker with cognitive actions.

Figure 4 shows the CC main blocks. The reaction of a CC to stimuli is called a *cognitive action*; in our system, it refers to a sampling policy. The CC selects a policy from the pool of exploration-exploitation policies based on the current mobility state, and then, the Sampling Decision Maker *instantiates* such policy using the predictions from the working memory. The cognitive action is kept in the executive memory to account for its execution in further PAC loops. The CC is expressed as:

(2) $$CC:\;\left(\rho ,\rho_{\mathrm{mismatch}\;\mathrm{flag}}\right)\mapsto S_{\mathrm{conf}}$$

where ρ and $\rho_{\mathrm{mismatch}\;\mathrm{flag}}$ are the prediction and mismatch flag, respectively, $S_{\mathrm{conf}}=\left(s,T_{\mathrm{real}}\right)$ is the sensor configuration, and s∈{GPS,accelerometer} and $T_{\mathrm{real}}$ the sampling.

### 4.1. Exploitation and Exploration Behavior

Mobility event changes cause the CC to select an exploitation or exploration policy:

- **Exploitation policies**: use a sampling that exploits the PRM’s predictions when mobility uncertainty is low, i.e., they are associated with *reducing energy consumption* actions suitable when the user roughly moves as expected.
- **Exploration policies**: are for cases on which predictions are not held, i.e., the CDS does not provide enough spatio-temporal accuracy due to mobility mismatches, and the CC tries to regain tracking accuracy based on the current mobility mode. Thus, these policies are associated with *reducing system uncertainty*.

The following section describes in detail the sampling policies designed for the CC, as well as the thorough process to generate cognitive actions for power savings.

### 4.2. Policies Tailored to Mobility States

We defined three sampling policies as cognitive actions. The stay point and trajectory state policies are for regular mobility ($S_{\mathrm{SP}}$ and $S_{\mathrm{trajectory}}$, respectively). The about exit policy is selected during the $S_{\mathrm{about}\;\mathrm{exit}}$ state (the user has not left the place at the predicted time). For the $S_{\mathrm{about}\;\mathrm{enter}}$ state, the trajectory state policy is selected because the user is still moving toward a stay point, and the accuracy of the tracking must be ensured.

#### 4.2.1. Stay Point State Policy

**Figure 5:**
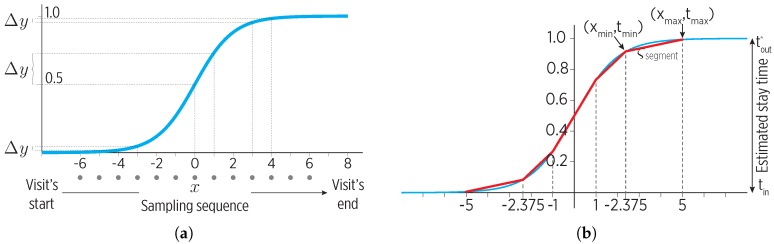
A representation of the sigmoid sampling policy for the $S_{\mathrm{SP}}$ state. (**a**) The non-linear increments (Δy) of the sigmoid suit the fast sampling rate at the start and end of a visit and the slow sampling at the middle. (**b**) A piece-wise linear approximation of the sigmoid so as to avoid long empty sampling intervals.

The motion state transition during visits could be modeled by a sigmoid function, $sig\left(x\right)=\frac{1}{1+e^{-\alpha x}}$. Note from Figure 5a that the sigmoid non-linearity can be exploited to map a sampling sequence with a fast rate during a visit’s start and end, and a slower sampling rate i the middle. The sigmoid generates the sampling during the $S_{\mathrm{SP}}$ state, using the stay times of the predictions provided by the working memory.

We approximate the sigmoid through $\varsigma_{n\;\mathrm{segments}}$ line segments, as shown in Figure 5b:

(3) $$\begin{array}{c} \Sigma_{\mathrm{segments}}\to \varsigma_{\mathrm{segment}}{\left(x_{min},x_{max},t_{min},t_{max}\right)}_{k},\forall \;k\in \;\left[1,\ldots ,\varsigma_{n\;\mathrm{segments}}\right] \end{array}$$

where $x_{min}$, $t_{min}$ are the domain’s start and timestamp, as $x_{max}$, $t_{max}$ are for the domain’s end. For each $\varsigma_{\mathrm{segment}}$, a specific $\varsigma_{\mathrm{time}\;\mathrm{sep}}$ time threshold indicates the maximum time gap without sensor data, e.g., minutes. The list of time thresholds defines the set $\Sigma_{\mathrm{time}\;\mathrm{seps}}$.

The sigmoid-based sampling produces a set $T_{\mathrm{function}}$ of intervals that are evaluated under the sigmoid to obtain the actual $T_{\mathrm{real}}$ sampling. The predicted stay time helps to map the sampling times from the sigmoid ($T_{\mathrm{function}}$) to real-world values ($T_{\mathrm{real}}$) according to the mobility learned from the user.

A different degree of spatio-temporal accuracy can be achieved through the $\Sigma_{\mathrm{segments}}$ and $\Sigma_{\mathrm{time}\;\mathrm{seps}}$ parameters, with a different energy impact. Although it could be simpler to divide the sigmoid into an arbitrary number of slices (i.e., linear space), defining such a value is hard: a large value would produce a fast sampling rate at the cost of consuming more energy, while a small value would reduce the energy consumption, but also the accuracy.

#### 4.2.2. Trajectory State Policy

There are many factors affecting the user’s mobility during a trajectory: meteorological phenomena, roads maintenance, etc., could cause discrepancies from expected mobility. We argue that while it is safe to rely on learned mobility to adjust the GPS sampling during visits, it is not the case in trajectories due to such discrepancies. As a result, we do not model motion during a trajectory other than the user moving within a speed range.

**Figure 6:**
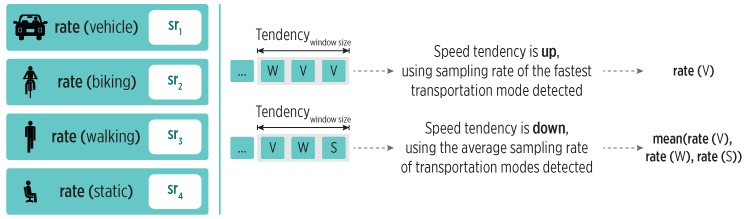
The sampling policy for the $S_{\mathrm{trajectory}}$ state. The speed tendency is inferred from a window of transportation modes, and a sampling rate is assigned accordingly. Sampling rates are related to the speed of typical transportation modes (the ${\mathrm{sr}}_{1}$ is the fastest one).

To this end, we employ the transportation mode detected by the HAR module to obtain a clue about the current user’s speed. As shown in Figure 6, we use a window of detected transportation modes (of length ${\mathrm{trj}}_{\mathrm{win}}\;\mathrm{size}$) to calculate a speed tendency as follows. We detect an *up* speed tendency when faster transportation modes are at the window’s end; if slower transportation modes are at the end, we detect a *down* speed tendency.

Each transportation mode is associated with a specific sampling rate. The sampling policy uses the detected speed tendency as follows:

- **Up speed tendency**: We select the sampling rate of the fastest transportation mode in the window. This aims to reduce the accuracy loss during location tracking.
- **Down speed tendency**: We select the average sampling rate of transportation modes in the window. This aims to smooth the decrements in the sampling rate, as the user might start moving again soon.

This policy uses both exploration and exploitation, as it quickly reacts to the increasing speed during a trajectory, while it also reduces the sampling rate during stops.

#### 4.2.3. About Exit State Policy

**Figure 7:**
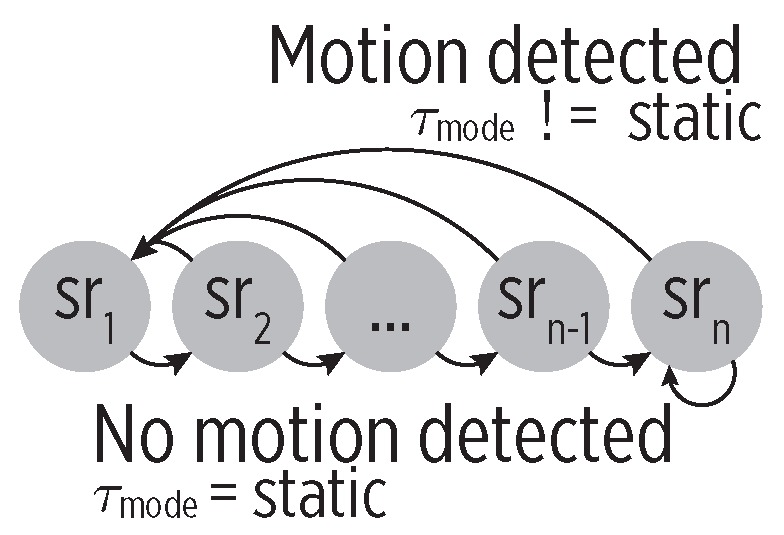
The sampling policy for the $S_{\mathrm{about}\;\mathrm{exit}}$ state. The HAR-based motion detection controls the transitions to adjust the sampling rate. The lack of motion causes the transitions from the fast-rate initial states (${\mathrm{sr}}_{1}$) to the slow-rate ending states (${\mathrm{sr}}_{n}$).

During the $S_{\mathrm{about}\;\mathrm{exit}}$ state, the CC aims to detect an eventual departure from the current stay point. To do so, the CDS checks user’s mobility via the HAR module as follows. As shown in Figure 7, we define a Finite-State Machine (FSM) with the set $\left\{sr_{1},\ldots ,sr_{n}\right\}$ of sampling rates as states (specifically, {30, 60, 90, 120, 150, 180 s}, although it is configurable). The FSM moves to the next state when motion is not detected (i.e., the outcome of the HAR module is static). Whenever motion is detected, the FSM moves back to the initial state and instructs a GPS sampling to confirm the place exit.

### 4.3. Fusion of Mobility Data Sources

**Figure 8:**
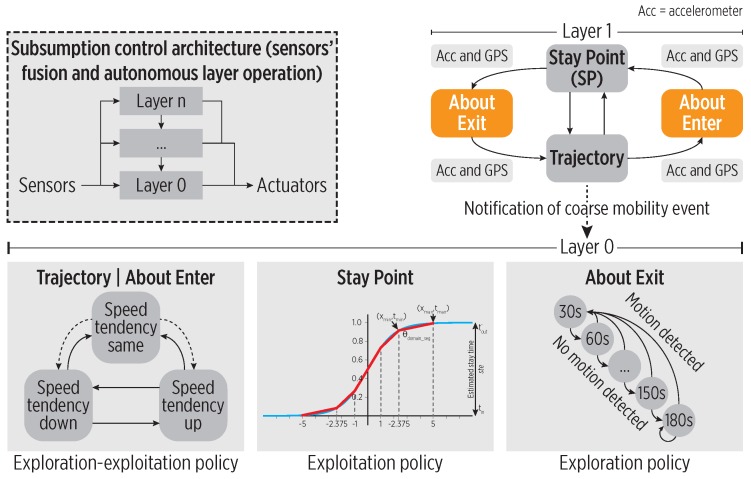
The subsumption architecture for the CC. The bottom layer includes different policies, executed according to detected transitions in the top level states.

As a framing architecture to implement the cognitive controller, we used the Subsumption Architecture (SA) proposed by Brooks [29]. The SA naturally couples sensory information to action selection in a layered hierarchical manner inspired by principles of brain organization [30]. The SA relies on distributed and parallel control, by integrating the perception, control, and action in a manner similar to animals: behaviors are decomposed into sub-behaviors organized into a hierarchy in which higher layers use lower ones to create a more complex behavior; see Figure 8. There is not a centralized control within these modules, but they communicate with each other via inhibition/suppression signals.

The CDS relies on combined fine- and coarse-grained mobility information derived from accelerometer and GPS data, respectively, to track the user with accuracy and energy efficiency.

Figure 8 shows the SA-enabled controller consisting of (i) a reactive control layer where low-complexity sensory events trigger simple actions or policies, (ii) an adaptive control layer that generates conditioned responses by exploiting the acquired representations as a result of learning, and (iii) a contextual control layer that generates specific actions when a discrepancy between expected and actual events occurs. In this sense, the cognitive map partially solves the lack of memory and symbolic representation in the SA. Moreover, the CC aids in the fusing of GPS and accelerometer data using two layers with distinctive and autonomous operation according to the active policy.

## 5. CDS Implementation and Experimental Evaluation

In this section, we discuss the implementation of our CDS on real-world devices, as well as its performance evaluation in both energy consumption and the spatio-temporal accuracy of detected events. We compared such performance against representative approaches in the literature, as well as against variants of itself (in terms of the sensors employed for mobility information input).

### 5.1. CDS Implementation

In this work, our solution was implemented on Android-powered mobile devices (Version 7, also known as Nougat) due to its flexible developing environment for accessing and controlling sensors’ information. However, the underlying principles of the proposed cognitive controller are still valid for other mobile platforms. The continuous and extended acquisition is hindered by power-saving mechanisms controlling the power-states at the system and application level: *Doze* and *App StandBy* features, respectively [31]. We implemented the following workarounds. We relied on the scheduling of Alarms to ensure that the mobile device was active (even woken up) during the sampling specified by the cognitive action. We implemented the CDS as a foreground service for whitelisting it to avoid its interruption by the OS. Similarly, we employed WakeLocks to ensure that the device remained active during data collection and processing.

As a way to enhance the GPS access provided by Android, we implemented a collecting interval (using a Timer and TimerTask combination); if a fix was not obtained, the perceptors were notified using a special event. Note that Android lacks ways to cancel location requests that are not likely to be obtained, leading to unnecessary energy overhead.

**Figure 9:**
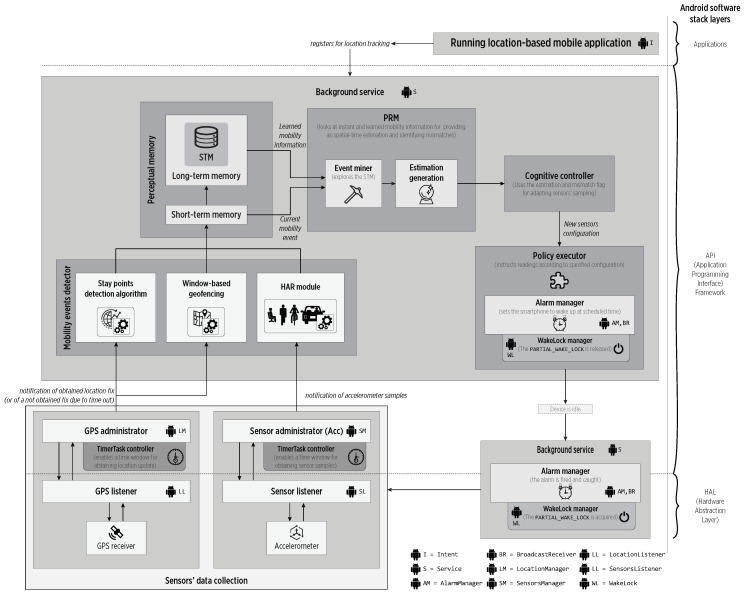
The CDS architecture implemented in the Android software stack. The different employed Android API components are labeled.

Figure 9 shows the CDS blocks implemented as a middleware using different Android API components across multiple OS layers for isolating the complexity of sensors access and power management. Here, the SA eased the implementation of the data fusion scheme and the nested FSMs shown in Figure 8.

For a fast system simulation and evaluation, we also implemented the CDS core components in a Python desktop environment. This desktop version uses files with timestamped locations as input, allowing a fast execution of experiments. The CDS requested timestamped location fixes, while a time-aware file reader delivered the proper fix in the file.

### 5.2. Materials and Methods (Setup)

We evaluated the CDS via desktop simulations and on-device deployments. We created a comparison baseline using location data collected with a 1-Hz sampling rate GPS logger (QStarz GPS logger device (Qstarz International Co., Ltd., Taipei, Taiwan), Model BT-Q1000EX. Additional information is on the official website [32]) and smartphones with a 30-s sampling rate. We processed such data using the CDS desktop version to detect mobility events and construct the ground truth cognitive maps, which were validated by participant users. For comparison, we used the output events detected by the CDS with different configurations, whether in desktop or on-device trials.

We used smartphones, with the specifications in Table 2, to collect mobility data of five users during several weeks. User 1 carried out the GPS logger together with a smartphone so that similar GPS signal strengths were observed. In total, we collected 17 trajectories, 8 of them with smartphone plus GPS logger data, while 9 of them only with smartphone data.

### 5.3. Spatio-Temporal Accuracy of Mobility Events

First, we evaluated the spatio-temporal accuracy of detected mobility events using different configurations of the $S_{\mathrm{SP}}$ state policy, as it produces the largest reduction in the GPS sampling rate with an impact on event detection accuracy. For simplicity, we implemented the policies for the $S_{\mathrm{trajectory}}$ and $S_{\mathrm{about}\;\mathrm{exit}}$ as fixed sampling rates. We launched experimental trials using the parameter values shown in Table 3, empirically selected, aiming at regular mobility scenarios.

**Figure 10:**
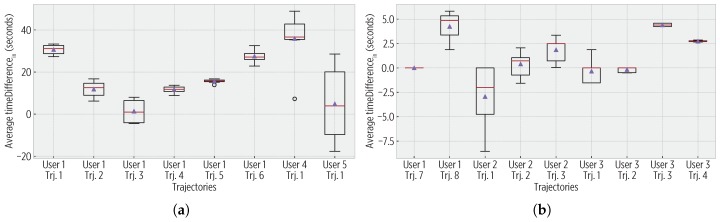
Enter time differences (${\mathrm{timeDifference}}_{\mathrm{in}}$). (**a**) In the 1-Hz trajectories, the largest difference being below 65 s. (**b**) For the 30-s trajectories, there are shorter differences (even sometimes negative), as for many cases, the same GPS fix in the ground truth and observed mobility triggered the detection of $ev_{\mathrm{in}}$ enter events.

**Figure 11:**
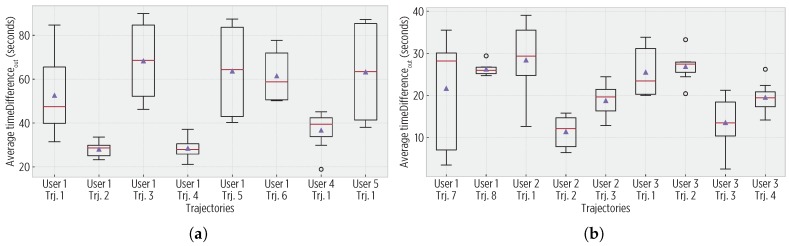
Exit time differences (${\mathrm{timeDifference}}_{\mathrm{out}}$). (**a**) For 1-Hz trajectories, values are within 65 and 165 s, aligned with the parameters of the sigmoid sampling (a theoretical maximum of 360 s). (**b**) For 30-s trajectories, the differences are shorter than in the 1-Hz trajectories due to reduced ground truth data.

For both 1-Hz and 30-s trajectories, all of the stay points were detected, and users confirmed this. Figure 10 shows the average enter time difference, which was short as the CDS used a trajectory policy when the users arrived at stay points. Similarly, Figure 11 shows the exit time difference. Although the sigmoid reduces the sampling rate, it does not produce a significant impact on the temporal accuracy, yet it reduces the energy consumption.

We measured the accuracy of the CDS during trajectories using the synchronized Euclidean distance [33]. For 1-Hz trajectories, a distance of 9 m was achieved. For 30-s trajectories, 273 m were obtained. Such accuracy depends on the user speed when moving, and the trials represent the worst scenario, as users were moving by vehicle.

### 5.4. Energy Savings

We also estimated the energy savings of our CC’s adaptive sampling, considering a constant energy consumption per GPS fix.

**Figure 12:**
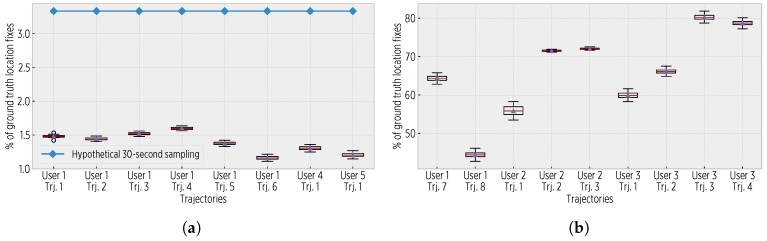
The proportion of saved location fixes. (**a**) For the 1-Hz trajectories, the CDS used an average of 1.42% of the ground truth fixes and 43% of a hypothetical 30-s sampling. (**b**) For the 30-s trajectories, the CDS used an average of 65%. Notice that the sigmoid configurations with the largest $\Sigma_{\mathrm{time}\;\mathrm{seps}}$ values used the lowest number of fixes.

Figure 12 shows the proportion of saved (avoided) location updates with respect to the complete location traces. The CDS requires significantly less fixes than those in ground truth traces (an average of 1.42%); due to the reduced size of ground truth data in the 30-s trajectories, this proportion is increased.

In fact, we also performed simulations with our CDS and a subset (32 trajectories) of the *cabspotting* database, which is composed of location fixes recorded up to 90 s apart by taxi cabs during 23 days [34]. For this dataset, our CDS employed 67% of ground truth fixes. (In consideration of its length (1,987,200 s), our CDS employed 0.71% of the cabspotting database). Although the presented results are based on simulations, they demonstrate the possibility of reduced energy consumption in accurate location tracking.

### 5.5. Comparison against Existing Approaches

We further investigated the actual CDS on-device performance with respect to other approaches, replicating the underlying principles of similar solutions and implementing two approaches as separated mobile sensing applications:

- Agnostic GPS sampling: It only collects location data using a 30-s sampling rate. No data processing (mobility inference) or sampling control is performed.
- HAR-assisted GPS sampling (Senseless-like): It triggers a GPS sampling whenever fine-grained motion is detected by the HAR module, as proposed by the *Senseless* framework [21]. The HAR module uses a 30-s sampling rate.

We used the parameter values shown in Table 4 for all on-device deployments, based on the results of desktop simulations. We deployed our CDS and one of the mobile applications on two Nexus 6 smartphones. The devices were carried together by a campus student, which was also given the logger device to collect ground truth data.

**Figure 13:**
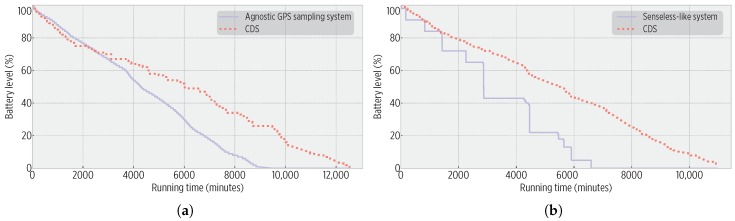
Smartphone batteries’ burnout for the CDS and existing approaches. (**a**) For the *Agnostic GPS sampling system* trial, the CDS increased the battery life by a factor of 1.3 (0.23 energy gain). (**b**) For the *Senseless-like* implementation, the CDS increased the battery life by a factor of 1.6 (0.53 energy gain).

Figure 13 shows the battery burnout of smartphones during the two trials. The CDS outperformed the other approaches after the first day; the most important places (home and work) were learned so that the CC adjusted the GPS sampling rate for energy efficiency. Table 5 and Table 6 details the sensors’ accesses for both trials. The poor energy performance of existing approaches was caused, for the agnostic GPS sampling, by its heavy use of GPS, while for the Senseless-like implementation, because of its frequent processing of accelerometer data to detect idleness. The unnecessary access to sensors and the processing of incoming data were lessened to some extent by our CDS, which improved the energy consumption.

Table 7 and Table 8 show the CDS spatio-temporal accuracy during these trials; the CDS accurately detected mobility events. Existing approaches outperformed the CDS in terms of the trajectory distance (12.66 m for the agnostic and 39.76 m for the Senseless-like system), at the cost of a higher energy consumption and the lack of mobility learning features.

### 5.6. Comparison against CDS Variants

We evaluated the CDS capability to use different input sensors:

- *Pure GPS CDS*: The CDS exclusively uses GPS data to derive fine-grained mobility (i.e., the transportation mode) based on traveled distance and elapsed time.
- *WPS CDS*: The CDS employs the WPS location provider rather than GPS. The lack of WPS data was associated with movement (i.e., no access points during trajectories) and its existence with idleness (i.e., user inside a building).

**Figure 14:**
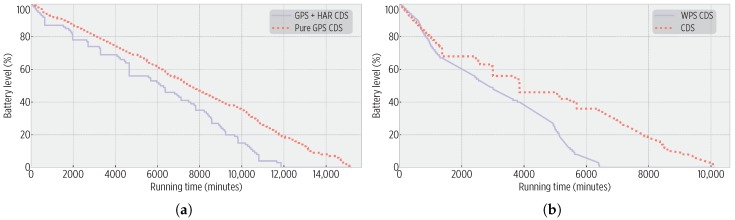
Smartphone batteries’ burnout: (**a**) For the *GPS + HAR CDS* vs. *Pure GPS CDS* trial; the *Pure GPS CDS* increased the battery life by a factor of 1.2 (0.26 energy gain). (**b**) For the *GPS + HAR CDS* vs. *WPS CDS*; the *GPS + HAR CDS* increased the battery life by 1.57 factor (0.39 energy gain).

Figure 14 shows the battery burnout during these trials. *Pure GPS CDS* achieved a better energy performance, partially due to the lack of accelerometer data collection and processing. On the right, the CDS (with HAR and GPS data) outperformed *WPS-CDS*, due to the high energy consumption of the Wi-Fi receiver to establish an Internet connection and determine the device’s location. Table 9 and Table 10 details the sensors’ accesses. On Table 9, notice the considerable number of HAR interventions that our CDS could perform, at the cost of the aforementioned shorter running time. On Table 10, see that *GPS + HAR CDS* could retrieve almost double the location fixes as *WPS-CDS*.

Such a balance of sensors’ accesses translates to the spatio-temporal accuracy shown in Table 11 and Table 12. Note on Table 11 that although the *Pure GPS CDS* consumed less energy than the *GPS + HAR CDS*, it was at the cost of a reduced accuracy in exit events’ detection: the HAR aided the CDS in detecting an eventual exit that was confirmed using the GPS. On Table 12, the impact of the WPS is observed as stay points and exit events were detected with a higher accuracy, which was caused by the short range of access points’ signal: the coordinates of retrieved fixes are very close to the access point (hence the short centroid distance), and as soon as the signal is lost, the departure is determined. More work is needed to define a combination of WPS-GPS that improves the CDS energy efficiency and ensures spatio-temporal accuracy.

## 6. Conclusions

We proposed a cognitive controller with event-based processing for energy efficiency and spatio-temporal accuracy in smartphone-based LBSs and MBSs. Our cognitive controller, part of a CDS architecture that learns mobility patterns from users, aims to reduce the information gap that a CDS has of its environment, which in our case was around the spatio-temporal predictions generated from learned mobility and the exploitation-exploration reactions implemented whenever such predictions were followed or missed. Our approach runs fully on-device, and we experimentally validated its accuracy and energy efficiency.

Overall, our CDS contributes to a more proactive role of the smartphone, particularly for LBSs and MBSs, providing the smartphone with true self-learning and self-adaptation, fundamental features for the design and deployment of cognitive systems in IoT. We aim to further investigate increasing the reliability of detected events by integrating more mobility data sources (sensors) using the SA control architecture. Similarly, we aim to explore the self-definition and adjustment of sampling policies using reinforcement learning and the energy-accuracy implications of decisions made. Finally, we aim to improve the efficiency of existing perception-action systems using the cognitive paradigm, in particular the memory and decision-making components. 

## Figures and Tables

**Table 1:** Mobility states defined for the proposed CDS.

State	Type	Description
Trajectory ($S_{\mathrm{trajectory}}$)	Observed	The user is moving between stay points, consistent with learned information.
Stay point ($S_{\mathrm{SP}}$)	Observed	The user is inside a stay point, consistent with learned information.
About Exit ($S_{\mathrm{about}\;\mathrm{exit}}$)	Derived	The user is inside a stay point, although she/he is expected to be already outside the place, i.e., there is inconsistency with respect of learned information.
About Enter ($S_{\mathrm{about}\;\mathrm{enter}}$)	Derived	The user is moving between stay points, although she/he is expected to be already inside a place, i.e., there is inconsistency with respect of learned information.

**Table 2:** Main technical specifications of the employed smartphones.

Feature/Smartphone	Motorola Nexus 6	Samsung Galaxy A5
System on Chip (SoC)	Qualcomm Snapdragon 805	Exynos 7580 Octa
Processor	Quad-core Krait 450 2.7 GHz	Octa-core Cortex-A53 1.6 GHz
GPU	Adreno 420	Mali T720MP2
Memory (internal storage)	3 GB RAM (64 GB)	2 GB RAM (16 GB)
Battery capacity	3220 mAh	2900 mAh
Battery technology	Lithium polymer (Li-Po)	Lithium-ion
Android OS	Nougat (7.1)	Nougat (7.0)

**Table 3:** Input parameters for the CDS spatio-temporal accuracy assessment.

**Cognitive** **Controller**	**Sigmoid segments (** $\Sigma_{\mathrm{segments}}$ **):**	(−5,−2.375),(−2.375,−1),(−1,1),(1,2.375),(2.375,5)
		(−4,−2.375),(−2.375,−1),(−1,1),(1,2.375),(2.375,5)
	**Time separations (** $\Sigma_{\mathrm{time}\;\mathrm{seps}}$ **):**	[90,150,180,150,90] seconds
		[90,120,180,120,90] seconds
		[60,150,180,150,60] seconds
		[60,120,180,120,60] seconds
	**Trajectory sampling**:	30-s fixed sampling rate
	**About exit sampling**:	60-s fixed sampling rate

**Table 4:** Input parameters for the on-device evaluation of the CDS.

**Cognitive** **Controller**	**Sigmoid segments (** $\Sigma_{\mathrm{segments}}$ **):**	(−5,−2.375),(−2.375,−1),(−1,1),(1,2.375),(2.375,5)
	**Time separations (** $\Sigma_{\mathrm{time}\;\mathrm{seps}}$ **):**	{60,120,180,120,60} seconds
	$S_{\mathrm{trajectory}}$ **sampling rates:**	Vehicle: 30 s, Biking: 60 s, Walking: 150 s, Static: 210 s (for speed tendency processing), ${\mathrm{trj}}_{\mathrm{win}\;\mathrm{size}}=3$
	$S_{\mathrm{about}\;\mathrm{exit}}$ **sampling rates:**	{30, 60, 90, 120, 150, 180} s (for the FSM based on motion detection)

**Table 5:** Sensors accesses for the *Agnostic GPS sampling* trial. The ground truth consisted of 720,227 fixes for the CDS and 535,494 fixes for the agnostic system.

	HAR Interventions	Requested Fixes	% of Ground Truth Fixes
*GPS agnostic* *sampling*	-	17,800	3.32
CDS	7090	6639 (5213 at GPS agnostic end)	0.92

**Table 6:** Sensors accesses for the *Senseless-like* trial. The ground truth consisted of 670,233 fixes for the CDS, and 417,234 fixes for the *Senseless-like* system.

	HAR Interventions	Requested Fixes	% of Ground Truth Fixes
*Senseless-like* system	13,251	399	0.09
CDS	5671 (4593 at *Senseless-like* end)	3236 (2004 at *Senseless-like* end)	0.49

**Table 7:** The CDS spatio-temporal accuracy during the *Agnostic GPS sampling* trial. All the stay points were detected; the missed visits were few and short, and the enter-exit time differences were under thresholds.

Spatial		Temporal	
Detected stay points	3	Missed visits	0 of 20
Missed stay points	0	Average missed visits time	0
Average centroid distance (m)	16.28	Average ${\mathrm{timeDifference}}_{\mathrm{in}}$ (s)	15.33
Average trajectory distance (m)	65.40	Average ${\mathrm{timeDifference}}_{\mathrm{out}}$ (s)	29.97

**Table 8:** The CDS spatio-temporal accuracy during the *Senseless-like* trial. Again, all the stay points were detected; the missed visits were few and short, and the enter-exit time differences were under thresholds.

Spatial		Temporal	
Detected stay points	6	Missed visits	5 of 27
Missed stay points	0	Average missed visits time	33.60
Average centroid distance (m)	60.62	Average ${\mathrm{timeDifference}}_{\mathrm{in}}$ (s)	21.5
Average trajectory distance (m)	77.24	Average ${\mathrm{timeDifference}}_{\mathrm{out}}$ (s)	56.27

**Table 9:** Sensors’ accesses during the *GPS + HAR CDS* vs. *Pure GPS CDS* comparison. The ground truth consisted of 711,593 fixes for the *GPS + HAR CDS* and 910,837 fixes for the *Pure GPS CDS*.

	HAR Interventions	Requested Fixes	% of Ground Truth Fixes
*GPS + HAR CDS*	4747	2819	0.39
*Pure GPS CDS*	-	8077 6318 at *GPS + HAR* *CDS* end)	0.88

**Table 10:** Sensors’ accesses during the CDS vs. *WPS-CDS* comparison. The ground truth consisted of 606,422 fixes for the CDS and 385,169 fixes for the *WPS-CDS*.

	HAR Interventions	Requested Fixes	% of Ground Truth Fixes
*WPS-CDS*	4264	942	0.24
CDS	5351 (4219 at *WPS-CDS* end)	1888 (995 at *WPS-CDS* end)	0.31

**Table 11:** Spatio-temporal accuracy for CDS variants during the vs. *Pure GPS CDS* trial. A single stay point was missed (it was at the edge of the parameters for stay points’ detection). The missed visits were few and short, and the enter-exit time differences were under thresholds.

Spatial			Temporal		
	*GPS + HAR CDS*	*Pure GPS CDS*		*GPS + HAR CDS*	*Pure GPS CDS*
Detected stay points	6	7	Missed visits	3 of 37	7 of 46
Missed stay points	1	0	Avg missed visits time	24.33	36.71
Avg centroid distance (m)	34.15	32.66	Avg ${\mathrm{timeDifference}}_{\mathrm{in}}$ (s)	26.26	33.10
Avg trajectory distance (m)	74.83	43.83	Avg ${\mathrm{timeDifference}}_{\mathrm{out}}$ (s)	43.47	71.10

**Table 12:** Spatio-temporal accuracy for CDS variants during the vs. *WPS-CDS* trial. Again, the missed visits were few and short, and the enter-exit time differences were under thresholds.

Spatial			Temporal		
	CDS	*WPS-CDS*		CDS	*WPS-CDS*
Detected stay points	3	3	Missed visits	1	0
Missed stay points	0	0	Avg missed visits time	51.00	0
Avg centroid distance (m)	46.02	10.01	Avg ${\mathrm{timeDifference}}_{\mathrm{in}}$ (s)	15.76	123.71
Avg trajectory distance (m)	60.16	-	Avg ${\mathrm{timeDifference}}_{\mathrm{out}}$ (s)	35.41	15.92
